# Virus and tumor microenvironment induced ER stress and unfolded protein response: from complexity to therapeutics

**DOI:** 10.18632/oncotarget.25886

**Published:** 2018-08-07

**Authors:** Kumari Asha, Neelam Sharma-Walia

**Affiliations:** ^1^ Department of Microbiology and Immunology, Chicago Medical School, Rosalind Franklin University of Medicine and Science, North Chicago, Illinois, U.S.A

**Keywords:** endoplasmic reticulum stress, UPR, PERK, IRE1α, ATF6

## Abstract

Endoplasmic reticulum (ER) stress can be activated by various pathological and physiological conditions including the unfolded protein response (UPR) to restore homeostasis. The UPR signaling pathways initiated by double-stranded RNA-activated protein kinase (PKR) like ER kinase (PERK), inositol requiring enzyme 1 α (IRE1α), and activating transcription factor 6 (ATF6) are vital for tumor growth, aggressiveness, microenvironment remodeling, and resistance to cancer therapeutics. This review focuses on the role of ER stress and activity of UPR signaling pathways involved in tumor formation and uncontrolled cell proliferation during various cancers and viral malignancies.

## INTRODUCTION

**Figure 1:**
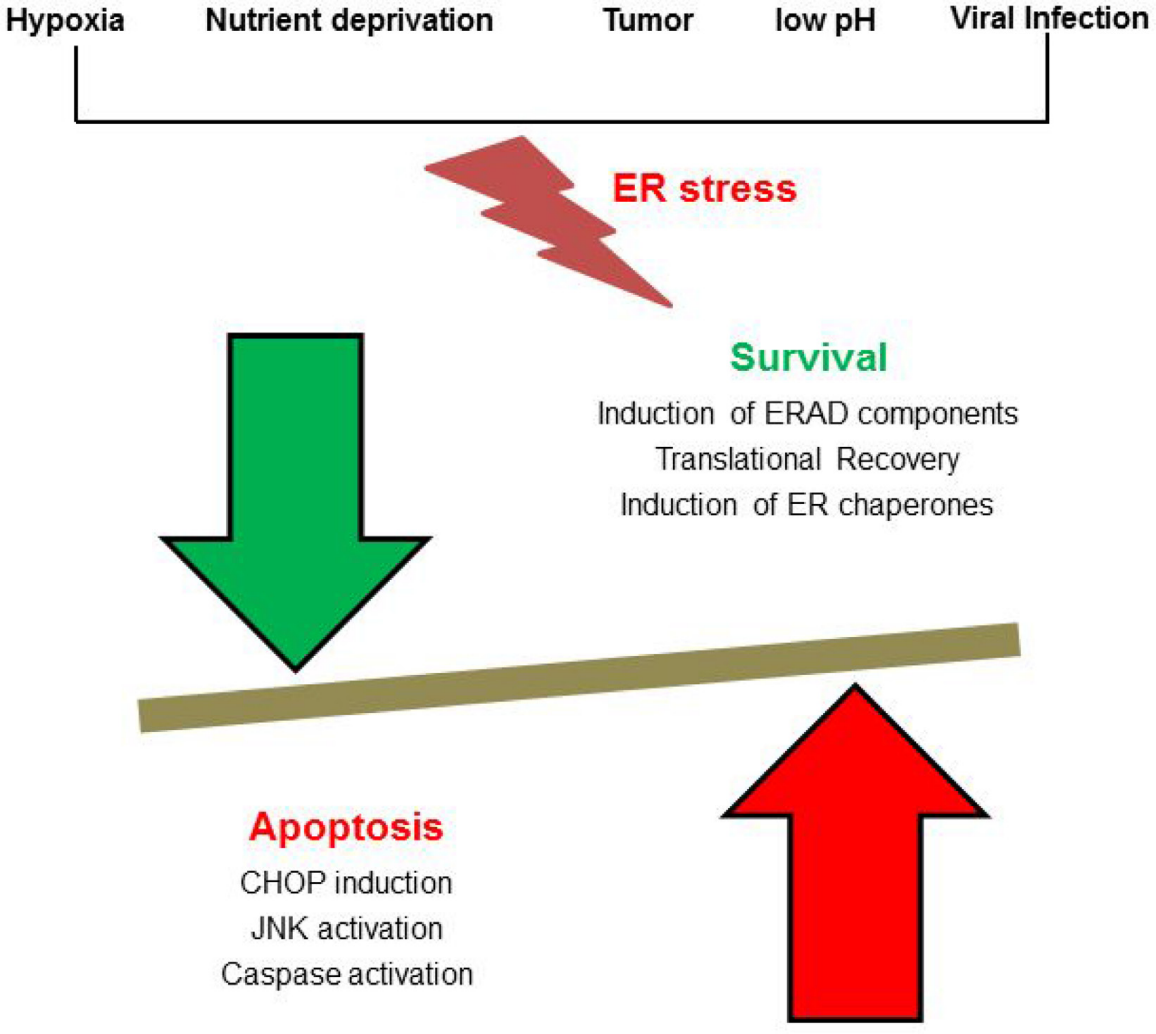
Players of ER stress: Cellular stress such as metabolite deprivation, hypoxia, cancer or viral infection causes an increased load of misfolded protein in the ER thereby triggering a stress response Either genes coupled with ER associated protein degradation get activated or translation of protein gets inhibited, and the cell dies.

Endoplasmic reticulum (ER) is the cell organelle that maintains cellular homeostasis and participates in lipid synthesis, protein folding, translocation, and post-translational modifications [1, 2]. Various stress factors like hypoxia, starvation, and change in pH, calcium depletion, and viral infection can disturb the ER environment (Figure 1). This disrupts the process of proper protein folding within the ER, finally leading to the accumulation of misfolded or unfolded proteins causing ER stress. It further activates “the unfolded protein response” (UPR), a cellular homeostasis response connecting the ER to the nucleus to restore cellular equilibrium [3, 4]. In order to repair ER-associated degradation (ERAD), UPR can activate apoptosis or degradation of unfolded or misfolded proteins, which cannot enter into the secretory pathway (Figure 1). Cancer cells and viruses have their own adaptive mechanisms to control ER stress-induced apoptosis, which allows them to grow aggressively. Here, we discuss various factors present in the tumor microenvironment of cancer cells, virus infected host cell responses such as UPR and ER stress, and their therapeutic repercussions.

### UPR signal pathways

**Figure 2:**
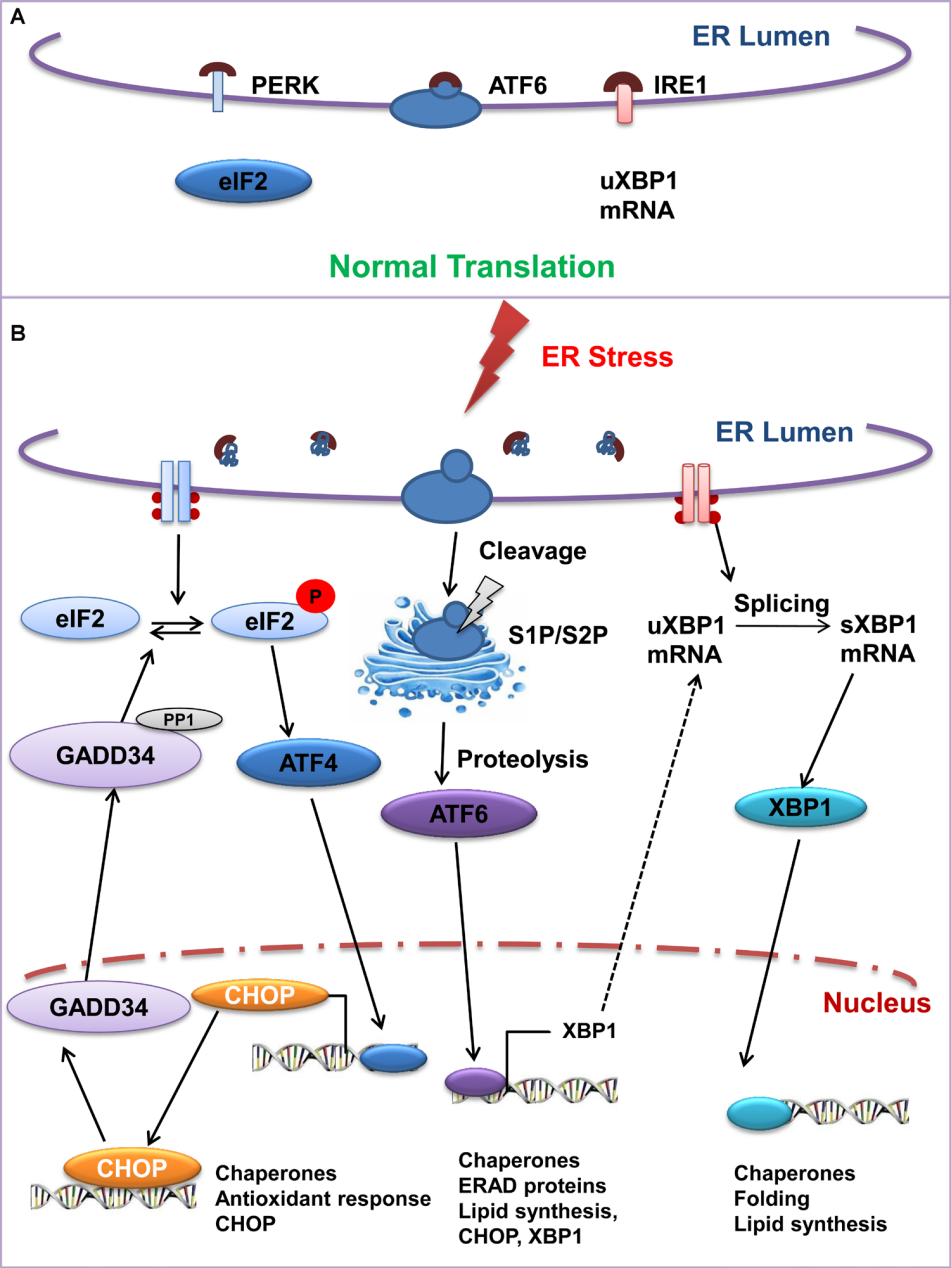
The unfolded protein response signaling cascade: IRE1, ATF6, and PERK serve as a UPR sensor under ER stress (**A**) In normal condition, BiP/GRP78 remains bound to these UPR sensors. But under (**B**) stress condition BiP/GRP78 dissociates from the UPR sensors thereby activating these signal transducers. Activated IRE1 facilitates splicing of XBP1 mRNA and spliced protein translocates to the nucleus to regulate ERAD. Likewise, cleaved transcription factor 6 (ATF6) induces the expression of ER chaperones and ERAD associated molecules. The activated PERK attenuates protein synthesis by phosphorylation of eIF2α, thereby enabling the translation of eIF2α-activating transcription factor-4 (ATF4), which translocates to the nucleus and induces the transcription of numerous genes.

In response to cellular stress, a programmed signaling cascade gets activated which is known as the unfolded protein response or UPR. It is mediated by three highly specific signaling protein molecules named activating transcription factor 6 (ATF6), double-stranded RNA-activated protein kinase (PKR)-like ER kinase (PERK), and inositol requiring enzyme 1 (IRE1) [5]. UPR is regulated by master regulator protein, Glucose-regulated protein GRP78 or Binding immunoglobulin Protein (BiP/GRP78) and GRP94 [5]. Under normal conditions, the ER luminal domain of these transmembrane molecules including ATF6, PERK, and IRE1 is bound to the chaperone protein BiP/GRP78, which maintains them in an inactive state [5]. Under stressful conditions (Figure 2), when misfolded or unfolded proteins accumulate in the ER lumen, BiP/GRP78 dissociates from these transmembrane sensors and activates UPR signal pathways and downstream target genes including ATF4, CHOP, ER degradation-enhancing alpha-mannosidase-like protein 1 (EDEM1), growth arrest- and DNA damage-inducible gene (GADD) 34, BiP/GRP78, GRP94, protein kinase inhibitor of 58 kDa (p58IPK), and PDI. Each of these pathways uses a different mechanism such as PERK inhibits translation, ATF6 regulates proteolysis, and IRE1 acts by degrading ER bound mRNAs [6].

Upon activation by ER stress, PKR-like ER Kinase PERK, oligomerizes and autophosphorylates its free luminal domain. The cytosolic domain phosphorylates the α subunit of translational initiation factor eIF2, and subsequently inhibits the process of translation. Thus PERK helps reduce the burden of unfolded protein. However, under limiting eIF2, translation of transcription factor ATF4 is induced. ATF4 is associated with two target genes: 1) C/EBP homologous protein (CHOP) and 2) growth arrest and DNA damage-inducible protein (GADD34). CHOP is a transcription factor that controls genes associated with apoptotic pathways. Thus the phosphorylation of eIF2α selects either a protective or an apoptotic role for PERK and is epitomized by the effects of alteration of its related phosphatases. GADD34; (a PERK inducible regulatory subunit of the protein phosphatase PP1c) dephosphorylates eIF2α and thus reverses the effect of PERK [7]. Deletion of GADD34 or inhibition of the GADD34-PP1c complex formation may be protective for cells (Figure 2).

IRE1 plays the dual role of a transmembrane kinase and endoribonuclease that participates in mRNA splicing to transmit the UPR signal. IRE1 undergoes conformational changes and oligomerizes in the ER membrane. Upon activation, IRE1 cleaves the uXBP1 mRNA encoding XBP1 (X-box binding protein 1), at two specific positions, excising an intron and finally giving rise to a spliced mRNA that is translated to the active form of XBP1 (Figure 2). XBP1 is a transcription factor that targets genes encoding ER chaperones, oxidoreductases, and ERAD pathway components. XBP1s plays a special role in regulating lipid biosynthetic enzymes, ERAD components as well as elements involved in active secretory pathways (Figure 2) [8]. Mammals have two IRE1 paralogues as IRE1α and IRE1β [9]. IRE1α is ubiquitously expressed and is required for XBP1 mRNA splicing [10]. IRE1β expression is restricted to the intestinal epithelium, and elimination of IRE1β in mice leads to increased signs of ER stress, increased JNK (c-Jun N-terminal kinase) signaling, and the mice are highly susceptible to experimental colitis induced by dextran sodium sulfate [11].

ATF6 is a membrane-bound transcription factor localized to the ER. ATF6 serves as both a sensor of ER stress and a transcriptional activator of UPR target genes [12]. Upon being activated by unfolded or misfolded proteins, ATF6 gets pinched off the ER and delivers the misfolded protein to the Golgi apparatus [13] where two proteases, Site-1 protease (S1P) and Site-2 protease (S2P) cleave [14, 15] and release N-terminal, ATF6 (N), that travels to the nucleus to activate UPR target genes. Among these targets are major ER-resident proteins involved in protein folding such as BiP/GRP78 and disulfide isomerase. These chaperones participate in refolding and relieving ER stress. The GRP78 promoter contains three ER stress response elements (ERSE) located upstream of the TATA element [16]. ATF6 is a member of the leucine zipper protein family that can constitutively induce the promoter of glucoseregulated protein genes through activation of ERSE [17]. ATF6 is also known to induce XBP1 expression and increase the sum of XBP1u and XBP1s [18]. All these three branches of UPR have opposing signals and their relative induction may shift the balance between cytoprotection and apoptosis. The complexity of regulation is further increased because the very components of UPR, including IRE1, XBP1, PERK, and ATF6, are themselves transcriptionally controlled by UPR [19].

### Tumor microenvironment

The tumor microenvironment is the cellular surroundings of the tumor, which includes both stromal and non-stromal components. Stromal cells secrete cytokines and growth factors and interact with tumor cells in reciprocal manner. Tumor cells affect stromal cell phenotype by modifying microenvironment. Stromal cells support tumor cell growth by promoting invasion and metastasis. The stromal components in tumor microenvironment include endothelial cells, immune cells, inflammatory cells, lymphocytes, and cancer associated fibroblasts (CAFs), the extracellular matrix (ECM) and signaling molecules (Table 1). Activated CAFs in the tumor microenvironment not only helps tumor cells proliferate by supplying nutrition but also secretes various growth factors (hepatocyte growth factor- HGF, fibroblast growth factor- FGF) and cytokines. CAF releases a large number of mesenchymal transducing soluble factors to remodel the ECM [20]. Endothelial cells lining tumor blood vessels produce pro-angiogenic factors and also promote migration, metastasis, and evade anoikis [21, 22]. Immune cells in the tumor environment may either activate CD4 helper and CD8 cytotoxic T lymphocytes in an MHC I and MHC II dependent manner or promote tumor growth in the presence of infiltrating leukocytes. As stated previously in this section, the UPR not only affects the processes of angiogenesis, inflammation and host immune response but also mediates the signaling between tumor and non-tumor cells [22]. Cancer cells communicate their message into the tumor vicinity through a paracrine effect on myeloid cells using small molecules, ions, proteins, and nucleic acids. These myeloid infiltrating cells become immunologically tolerant and start secreting many signaling molecules such as growth factors, cytokines, and exosomes. UPR enriches tumor fitness by being transmitted from cancer cells to the cells of the tumor microenvironment. This transmissible ER stress (TERS) has multiple effects on the recipient cells *in vitro* and *in vivo* [23]. TERS upregulates tumorigenic inflammatory cytokines (IL-6, IL-8, IL-23), inflammatory metabolite PGE2 and decrease T cell response [24]. TERS control immune cell development, function, and survival in both the pathological and physiological conditions.

**Table 1:** Tumor microenvironment

Cellular components	Non-cellular components
Cells of hematopoietic origin: cells that arise in the bone marrow – Lymphoid cells- T cells, B cells and NK cells Myeloid cells- Macrophages, neutrophils and myeloid-derived suppressor cells (MDSCs). Other cell types- Macrophages, platelets and dendritic cells.	Non-cellular components: Extracellular matrix (ECM) consisting of many distinct components — including proteins, glycoproteins and proteoglycans
Cells of mesenchymal origin: Includes fibroblasts, myofibroblasts, mesenchymal stem cells (MSCs) and adipocytes. Angiogenic cells- Endothelial cells and pericytes	Growth factors: Angiogenic growth factors: VEGF, FGF2 Epidermal growth factor (EGF) family- TGFα, EGF, AR Platelet-derived growth factor (PDGF) Cytokines and chemokines (CXCL12 and interleukin (IL-8) Osteoclastogenic growth factors- M-CSF, RANKL Cyclooxygenase-2 (COX-2) and prostaglandins

Besides carcinoma cells, the tumor microenvironment also consists of cellular components of myeloid and mesenchymal origin along with non-cellular components. A major non-cellular component in the tumor microenvironment is the extracellular matrix (ECM) that facilitates the maintenance of tumor structure and functionality. Abnormal ECM has been reported to promote tumor progression and angiogenesis.

The tumor microenvironment is comprised of hyperactive pro-oncogenic genes and mutant tumor suppressor genes that are responsible for the highly proliferative and metabolically demanding cellular environment [25]. Due to the metabolically demanding stress environment, tumor cells thrive in conditions of hypoxia, glucose deprivation, lactic acidosis, oxidative stress, alteration in nitrogen species, lipid peroxidation, and decreased amino acid supplies [25, 26]. Premalignant cells have the added advantage of gene mutations, which curb UPR induced apoptosis or senescence promoting survival and growth [27]. All these changes due to intrinsic and extrinsic factors of the microenvironment contribute to the UPR activation induced by ER stressors. Unlike normal cells, cancer cells demonstrate constitutive activation of the UPR system (IRE1α-XBP1) under stress conditions [28, 29]. IRE1α mutants lose their regulated IRE1-dependent decay of mRNA (RIDD) function and show increased cell survival [30].

### Hypoxia

The tumor microenvironment has poor microcirculation, which causes hypoxia in the cell. Hypoxia leads to UPR activation, which eventually initiates another cycle of cell proliferation and subsequent hypoxia in tumor cells [31]. Although hypoxia is not ideal for general protein translation but in the case of malignancy, hypoxia induces the upregulation of hypoxia-inducible factor 1 alpha (HIF1-α), which stimulates angiogenesis and activation of metastatic genes [32]. Hypoxia triggers PERK signaling in mammalian cell lines [33]. The UPR activation mediated by downstream effectors of eIF2α phosphorylation and activated IRE1 develops hypoxia tolerance [34]. Activation of hypoxic-modulated molecular responses depends on cellular oxygen levels and the duration of hypoxia. UPR mediated induction of endoplasmic reticulum oxidoreductin 1 (ERO1) [35, 36] leads to generation of reactive oxygen species (ROS) in mitochondria [37] and ER during hypoxia.

### Reactive oxygen species (ROS) and inflammatory mediators

ROS in the tumor microenvironment promotes genetic and epigenetic alterations favorable for tumor growth and progression [38]. ROS can target ER resident proteins and ER based calcium (Ca2+) channels leading to ER stress signaling. Increased cytosolic calcium and calcium ingress in mitochondria from ER stimulates further ROS production [39]. This process activates PERK mediated Nrf2 induction which promotes cancer cell proliferation [40]. Proinflammatory cytokines like IL6 and tumor necrosis factor α (TNFα) present in the tumor microenvironment further add to the ER stress induced UPR activation. All three branches of the UPR pathway activate NF-kB through different mechanisms. PERK-eIF2α signals translational arrest and increased NF-kB/IkB ratio, leading to NF-kB nuclear translocation [41, 42]. ATF6α activates NF-kB through AKT phosphorylation [43, 44]. IRE1α–TNF receptor-associated factor 2 (TRAF2) complexes can bind to NF-kB leading to IkB phosphorylation and degradation, and nuclear translocation of NF-kB [45]. The TRAF2-IRE1 complex initiates proapoptotic signaling by activating Apoptosis Signal Regulated Kinase (ASK1), which subsequently transmits the death signal to c-Jun N-terminal kinase (JNK). JNK phosphorylates Bcl2 and abrogates its anti-apoptotic activity [46].

Various inflammatory mediators, such as eicosanoids including prostaglandins and leukotrienes have also been associated with ER stress and UPR. An *in vivo* study carried out in a murine model showed that insulin-sensitizing effects of 5-LO siRNA or zileuton is due to LTB4 downregulation and AMPK activation [47]. LTB4 was identified as the critical player in the ER stress pathway, reactive oxygen species (ROS) generation and inflammation [47]. Glucose deprivation in colorectal cancer cells increased cycloxygenase-2; COX-2 and reduced 15-hydroxyprostaglandin dehydrogenase; 15-PGDH expression and thus upregulated extracellular inflammatory prostaglandin PGE2, which promoted cancer cell survival. These studies emphasized the role of inflammatory mediator PGE2 as mediator of cell survival during adaptation to the tumor microenvironment, which can lead to novel therapeutic strategies [48]. Another eicosanoid, leukotriene C4 (LTC4), has also been reported as a major ER stress mediator that is also observed in chemotherapy triggered oxidative stress, DNA damage and dsDNA breaks [49].

### Nutrient deficiency and angiogenesis

Tumor cells grow in a glucose deficient environment, which causes accumulation of misfolded protein within the ER affecting calcium concentration that activates PERK [50]. Tumor cells switch to a high rate of aerobic glycolysis producing lactic acid [51] and activate XBP1 and PERK/ATF4-mediated UPR components. BiP/GRP78 is also upregulated in a glucose deficient tumor microenvironment [52]. Amino acid deprivation induces eIF2α phosphorylation. Various growth factors like epidermal growth factor (EGF), transforming growth factor-α (TGF-α) released within the tumor microenvironment activates UPR. Wang and colleagues demonstrated that nutrient deficiency activates UPR in an IRE1α/XBP-1, PERK-ATF4, and ATF6α dependent manner and stimulates inflammatory cytokine (IL 6), fibroblast growth factor-2 (FGF-2), and vascular endothelial growth factor (VEGF) signaling [30, 53].

All these studies suggest that UPR activation in tumor cells is marked by both intrinsic and extrinsic factors. The high metabolic demand of the tumor microenvironment activates UPR and subsequently augments oncogenes or mutations in tumor suppressor genes and increases protein synthesis, and translocation into the ER. Additionally, cancer cells being secretory in nature are prone to UPR activation.

## ER STRESS COMPONENTS IN DIFFERENT TYPE OF CANCERS

Cancer cells differ from normal cells in their ability to manipulate ER stress induced cell death and are thus resistant to apoptosis. The three branches of UPR are associated with different phases of growing tumors. For example, IRE1 signaling plays a crucial role during hepatocellular carcinoma (HCC) initiation [54]. Likewise PERK signaling helps colorectal cancer cell and squamous cancerous cells to survive in a nutrient and oxygen deficient tumor microenvironment [55, 56]. All three UPR signaling transducers are involved in progression of prostate cancer [57]. The major UPR inducing pathway in tumor is mediated by hypoxia. Recent studies have shown that spliced XBP1, a major component of the IRE1 pathway, promotes cancer cell survival by forming a transcriptional complex around hypoxia-inducible factor-1 (HIF-1) [58]. In the case of breast cancer, HIF functions as a chief regulator by aiding in transcription of genes responsible for expressing proteins that are essential to metastasis. It also participates in the process of epithelial mesenchymal transition (EMT), invasion, injury, extravasation, and metastatic niche formation. ER stress drives EMT in *in vitro* and in animal models of fibrosis through src-mediated signaling and contributes to cancer cell invasion [59]. ER stress also plays an important role in ER-mitochondrial communication. Activation of the classical UPR of ER is necessary for mitochondrial proteotoxicity or mitochondrial UPR (UPR^mt^). Mitochondrial HSP90 chaperone and its related protein, TRAP-1, are abundant in the mitochondria of tumor cells but not in those of healthy tissues, and they appear to antagonize mitochondrial death pathways [60]. Impaired function of mitochondrial HSP90 leads to a mitochondrial UPR and the induction of autophagy [61]. HIF is also involved in the progression of triple negative breast cancer [58, 62]. XBP1 is also known to modulate endoplasmic reticulum lipid raft associated 2 (ERLIN2) protein expression, which possess the capacity to protect breast cancer cells from ERAD promoting their survival [63]. The estrogen-mediated increase in GRP78, in breast cancer cells expressing estrogen receptor α [NR3A1] confers improved resistance to ER stress and cell proliferation both of which can be decreased through siRNA mediated knockdown of estrogen receptor α [64]. Tamoxifen has been designed to block NR3A1, in estrogen receptor positive (ER+) breast cancer [65]. HER2-positive tumors have higher sensitivity to inhibition of HSP90 since HER2 is a client protein of Hsp90 [66]. Pancreatic ductal adenocarcinomas (PDACs), with an extremely poor prognosis of a one year survival, have been shown to become significantly hypoxic as they grow. Activation of both PERK and IRE1 arms of the UPR are delayed in the presence of ER stressors in pancreatic cancer cells as compared to normal pancreatic cells [67]. Furthermore, adapting to chronic ER stress has been related to the induction of anterior gradient 2 (AGR2) that contributes to the initiation and development of PDAC [68]. Along with chemotherapy, radiotherapy is another component of the mainstay treatment for cancers of lung, breast, and prostate. Addition of radiotherapy to a patient treatment plan adds to the complexity as it activates multiple cell signaling cascades and induction of ER stress. A better understanding is required to study the influence of UPR function on radio- chemo- and sensitivity in extremely mucinous and secretory breast cancers as these forms of cancers are more dependent on UPR. Pharmacological manipulation of downstream UPR pathways may improve and increase tumor cell killing and reduction of toxicity to the neighboring healthy tissue.

### Viral pathogenesis

Viral infection of host cell depends on competition between virus infection and the host response. The host cell, if conquered, gets acute or chronic infection. Each step in the viral life cycle beginning from viral entry and replication into the host cells to release of mature virions can be deleterious to host. Expression of viral protein, imbalance of calcium concentration by viroporins and consequent depletion of ER membrane due to the release of virions [69] cause ER stress in the host cell by generating loads of unfolded or misfolded proteins [1]. The host cell generates UPR to promote the ERAD pathway against viral infection. Viruses (RNA/DNA virus) may manipulate the host UPR in a way to maintain an environment favorable for its infectivity and persistence in the diseased cell (Table 2) [70, 71].

**Table 2:** Different RNA and DNA viral diseases and associated ER stress pathways

RNA Viruses	**ER stress signaling pathway**	**Virus - viral protein**
	PERK- eIF2α pathway	• Hepatitis C Virus- E2 and NS5A • Vesicular stomatitis virus -M protein • Japanese encephalitis virus -NS2A • Influenza A virus -NS1
	IRE1–XBP1–ERAD pathway	• Hepatitis C Virus - E1 and/or E2 • Hepatitis E Virus -ORF2 • Dengue Virus -DENV2
	ATF6 pathway	• Dengue Virus-DENV2 • Epstein-Barr virus -EV71 • Influenza A virus -HA
DNA Viruses	PERK- eIF2α pathway	• Hepatitis Simplex Virus 1- γ134.5 Protein • African swine fever virus- DP71L • Hepatitis B Virus - S protein • Papillomavirus- E6 • Vaccinia virus-E3L • Cytomegalovirus- pUL38
	IRE1–XBP1–ERAD pathway	• Hepatitis B Virus- HBx and S proteins • Cytomegalovirus-US11, pUL38

Among such chronic viruses is a family of herpesviruses, which utilize host UPR to maintain latency in the host. Herpesvirus mimics many host molecules and utilizes UPR to set up active lytic infection and to break dormancy of latent phase, thereby proving long time interaction of virus with host UPR. In the case of gammaherpesviruses, human herpesvirus 8 (HHV8) or Kaposi’s sarcoma associated herpesvirus (KSHV) studies have shown that primary effusion lymphoma (PEL) cells are immunophenotypically similar to pre-plasma cells and have unspliced XBP-1 mRNA [72, 73]. Under ER stress XBP-1 mRNA is processed to XBP-1s, which binds to the KSHV ORF50 promoter and upregulates KSHV ORF50 gene expression inducing KSHV lytic replication. In an elegant study, Paul M. Lieberman's group reported that the ER stress due to different stimuli triggers RAD21 cleavage and lytic switch in PEL cells [74]. They also found that few ER stress inducers such as DTT and protein synthesis inhibitors, were more effective in inducing RAD21 cleavage and lytic reactivation than others. For example, SubAB cytotoxin and tunicamycin, induced only partial cleavage of RAD21 with slower kinetics and lower amplitude of KSHV lytic reactivation. The slower kinetics of these compounds may be associated with a distinct ER stress induced by them when compared to broadly acting chemical inhibitors [75] which may be modulating additional pathways to activate a broader range of caspases resulting in complete cleavage of RAD21. PEL cells are sensitive to ER stress-inducing agents, like chloroquine and it can be linked to a defect in the PERK and IRE1α [76, 77]. This particular sensitivity of PEL cells may control KSHV latency epigenetically. Similar *in vitro* and clinical studies have shown that induction of ER stress by 2-Deoxy-D-glucose (2-DG) shuts down viral replication and lytic gene expression. Thus the study not only supports the therapeutic potential of glucose/mannose analogs against gammaherpesvirus but also suggests the possible usage of this approach against other virus-driven cancers. 2-DG downregulates mRNA levels of numerous viral genes required for replication, including regulator of transcription activator (RTA), thereby impairing RTA transactivation loop subsequently leading to the inhibition of KSHV lytic gene transcription cascade [78]. Our studies demonstrated that HHV-8 utilizes arachidonic acid (AA) pathway enzymes (cyclooxygenase-2; COX-2, 5-lipoxygenase; 5-LO, leukotriene A4 hydrolase; LTA4H) and its metabolites (prostaglandin E2_;_ PGE2, leukotriene B4; LTB4) in their life cycle, especially in the maintenance of its latency, and effective inhibition of these pathways could potentially be used in treatment to control KS/PEL [79–82]. Unpublished results from our lab demonstrate that KSHV infection induced LTA4H serves as a link between KSHV life cycle and its adaptation to the host cell ER stress. Use of Bestatin, a well-known LTA4H inhibitor or LTA4H silencing induced proliferation arrest in KSHV+ PEL cells when compared to non-infected (KSHV–; BJAB) Burkitt’s lymphoma cells. LTA4H inhibition induced ER stress proteins (calnexin, BiP, IRE1-α, and CHOP) and stimulated phosphorylation of PERK and eIF2α, which might be causing translation arrest and apoptosis in PEL. We also demonstrated that KSHV infection downregulates anti-inflammatory and pro-resolving metabolites such as lipoxins for successful viral life cycle and pathogenesis [83, 84]. It has been proven that Varicella-zoster virus (VZV), which possesses smallest genome of human herpesviruses, lacks a few genes used by other herpesviruses to manipulate the UPR [85]. Later, John Carpenter and Charles Grose established that VZV differentially induced the upregulation of UPR to deal with viral glycoprotein synthesis [86]. VZV upregulated cAMP responsive element binding protein H, an atypical UPR molecule [86]. Clearly, this will pave the way for future studies to disclose the relationship between VZV and UPR. Many viruses have evolved mechanisms to antagonize UPR induced eIF2α phosphorylation and translation control. Among these viruses is Herpes simplex virus I (HSV-I), which encodes ICP34.5, a protein homologous to cellular protein GADD34, that dephosphorylates eIF2α so that protein translation can proceed [87]. Likewise the African swine fever virus, encodes DP71L, which shows homology to HSV ICP34.5 protein and can also associate with the enzyme that dephosphorylates eIF2α [88].

Paradoxically, proteins that are expected to induce ER stress at low levels act as inhibitors of ER stress instead. For example, HCV and HSV-1 viral envelope glycoproteins E2 and gB work as specific inhibitors of PERK [89, 90]. ER stress and the UPR have been aiding in viral replication. Human cytomegalovirus US11 protein and herpesvirus US2 activate UPR to increase degradation of the class I major histocompatibility complex proteins [91] resulting in an escape from the host immune response. For many reoviruses competing with host mRNA molecules, increased ATF-4 expression induced by eIF2α phosphorylation is beneficial for viral replication [92]. The promoter of hepatitis B virus (HBV) is upregulated by ER stress [93]. Thus, it can be concluded that some viruses have evolved mechanisms to flee the negative effect of ER stress and concurrently exploit preferred UPR induced factors to their own advantage. In case of severe acute respiratory syndrome coronavirus (SARS-CoV) accessory viral protein binds to ATF6 domain thus inducing proteolysis of ATF6 [94]. The cleaved DNA binding and transcription activation domains of ATF6 then move from ER to the nucleus [94]. These findings suggest that viruses may exploit their own protein(s) to directly modulate UPR responses. Viruses can also exploit the ERAD pathway [95] to degrade immune molecules or viral envelope glycoproteins to escape immune responses of the host. Polyomaviruses use ERAD during their life cycles to uncoat the capsid and present the nuclear localization signals on capsid proteins for initiation of viral DNA replication and virion maturation. Viruses can hinder ERAD tuning and can use ERAD-associated machinery to form isolated lipid vesicles for replication and as an escape from immune detection.

UPR in the host is much more than a homeostatic cellular response to virus infections. UPR is closely associated with inflammatory pathways and innate immunity induced by host pattern recognition receptors (PRRs). As a response to this the professional virus killer gets activated and releases interferons. One of the proximal UPR sensors, inositol-requiring enzyme 1 (IRE1) which activates IRE1-dependent decay (RIDD), is evolutionarily related to virus killer RNase L [96]. Since viruses naturally infect and transfer DNA into the host cells, viral vectors are now being increasingly used as gene delivery vehicles in gene therapy. Vectors based on retroviruses integrate their viral genome into the chromosomal DNA of the host cell. Episomal vectors like adenovirus and HSV-1 deliver their genomes into the nucleus of the target cell, where they continue as separate extrachromosomal element. Vaccinia viral vectors have also been used for cancer gene therapy [97, 98] because of proficient infectivity and gene expression in complex tumors [99]. Also, the safety profile of Vaccinia virus has already been determined due to its use as a vaccine for small pox in humans [99]. Viruses from the *Herpesviridae* family (VZV, EBV) are also being used for cancer gene therapy [100]. EBV, owing to its specificity for B cells, has been utilized to deliver (granulocyte-macrophage colony-stimulating factor) GM-CSF therapy to patients of B-cell chronic lymphocytic leukemia (B-CLL) [101]. Since EBV and other viruses are infective to cells, their modification and validation is essential to make them safe enough to be used for gene therapy. It is becoming significantly important to study and understand virus host interactions so that viral vectors can be used in gene therapy [102].

## THERAPEUTIC STRATEGIES TO CONTROL ER STRESS RESPONSES IN CANCER

As discussed, stress stimuli in the tumor microenvironment activate UPR, which further influences cellular processes such as metastatic growth, apoptosis, inflammation, and host immune responses. There can be two strategic plans to target UPR signaling- one by effectively killing cancer cells or by hindering UPR-mediated adaptive responses which may help tumor cells proliferate in harsh tumor microenvironment conditions and resist the treatment.

UPR effectors upregulate VEGF-A and other pro-angiogenic factors in cancer cells [63, 103] including breast cancer [104, 105]. BiP/GRP78, a master regulator for ER stress response helps premalignant cells adapt to UPR induced apoptosis. So therapeutic strategies aimed at reducing BiP/GRP78 or targeting UPR sensors can be effective against cancer. BiP/GRP78 is increased in a variety of cancer types including breast, lung, colon, prostate, skin, melanoma and many other malignancies [64, 106, 107]. Our study and similar studies from the past reported high levels of GRP78 are associated with rapid proliferation and malignancy of tumors [64, 108]. In breast cancer cells associated with estrogen receptor α [NR3A1], increase in GRP78 abundance signifies cell proliferation and improved resistance to ER stress. The siRNA-mediated knockdown of estrogen receptor α can be strategically used to prevent tumor progression in these cases [64]. Similarly, chemotherapeutic agents can be used to reduce GRP78 in glioblastoma cell lines and solid tumors. The therapy increases expression of CHOP and caspase 7, leading to inhibition of tumor formation and finally inducing cellular apoptosis [108, 109]. BiP/GRP78 is positively regulated by the mitogen activated protein kinase MAPK pathway. Thus inhibition of the MAPK pathway boosts caspase-4 mediated ER stress induced apoptosis in melanoma cells. Moreover, there are antitumor agents that decrease GRP78 at the protein level by directly binding GRP78 to facilitate its degradation [108]. Based on these characteristics, GRP78 is considered as a biomarker of cancer progression [110]. Since inhibition of BiP/GRP78 can play a cytoprotective role, therefore it is being utilized in developing treatment strategies against multidrug resistant cancer. Likewise UPR signal transducers and proteasome inhibitors such as Bortezomib [111] involved in activating UPR can also be used to check cancer progression.

XBP1 represents a regulator of plasma cell differentiation and overexpression of XBP1 is critical for multiple myeloma induction. Inhibition of XBP1 splicing has been shown to reduce multiple myeloma cell growth. XBP1 splicing induced by IRE1α, prompt cellular proliferation through increased expression of cyclin A1 thus IRE1α inhibition may expose multiple myeloma cells to ER stress and reduce their survival [112]. Chevet *et al*, 2015 and other groups of scientists have shown that directed therapeutics targeting the RNase activity of IRE1 reduces the progression of various forms of cancer [113, 114]. Similar approaches have been pursued in various models of experimental glioblastoma [115–117]. Studies on human cancer tissue samples (Cancer Genome Atlas Research Network, 2008; Parsons *et al*, 2008) revealed the presence of three somatic mutations (S769F, Q780 and P336L) on the IRE1 gene in GBM [118, 119]. Chevet *et al*, 2018 also showed the antagonist role of XBP1s and RIDD on tumor growth, where RIDD of mRNA and miR17 depict anti-tumoral features by remodeling of tumoral stroma [120]. Recent studies have shown that STF-083010, an inhibitor for XBP1, can restore sensitivity to tamoxifen in tamoxifen resistant MCF-7 cell lines and a synergistic effect of both drugs significantly delay breast cancer progression. Clinical trials have further demonstrated that XBP1 expression levels are highly correlated to cell survival in ER+ breast cancer patients. Loss of XBP1 has been linked to spontaneous intestinal inflammation and inflammatory bowel disease in humans [121]. Modulators of IRE1 signaling like MKC-3946 [103], 3-methoxy-6-bromosalicylaldehyde [122], 4μ8C [123], STF-083010 [124], KIRA-6 and toyocamycin [125] are being therapeutically used in the case of multiple myeloma. These inhibitors are shown to kill cancer cells or sensitize them to common chemo- or radiotherapies. The inhibitors of XBP1 mRNA splicing work efficiently and do not affect the phosphorylation and the oligomerization of IRE1. ATF6 negatively regulates genes involved in cellular senescence and mediates cell survival through upregulation of LC3B, a component of the autophagosomal membrane. ATF6 is also responsible for upregulation of XBP1 and thus BiP/GRP78 expression in liver cancer [126].

Under hypoxic conditions the PERK/eIF2α pathway increases cell growth and survival of cancerous cells. Since PERK activates many angiogenic genes, its inhibition was found successful in reducing tumor growth both *in vitro* and *in vivo*. Recently our lab and other ongoing studies have found that Salubrinal is efficient in curbing dephosphorylation of eIF2α and thus can be used as a potential therapy against inflammatory breast cancer [127]. These results indicate that new therapeutic antitumoral agents inhibiting ER chaperones level or UPR adaptive and prosurvival pathways can be utilized as potential cancer therapies with improved outcomes. Recently, two anticancer drugs GSK2606414 [128] and GSK2656157 [129], which act as ATP competitive inhibitors and target PERK/eIF2α signaling based either on eIF2α phosphorylation inhibition or on its prolonged phosphorylation, have been introduced. Inhibition of eIF2α phosphorylation by PERK causes increased ER protein load and further reduces adaptation to ER stress leading to cell death. GSK2656157 has been reported to reduce tumor growth in mouse xenograft models [130]. Most recently the integrated stress response inhibitor (ISRIB) has been identified as a new PERK signaling inhibitor which rather than inhibiting PERK or eIF2α phosphorylation blocks downstream processes to reverse the effects of eIF2α phosphorylation. ISRIB treatment only affects the survival of cells under ER stress [131].

One of the major undesirable consequences of cancer therapy is that many anticancer drugs activate UPR that can lead to drug resistance in patients. Thus recent combined therapeutic approaches are proving to be more efficient against UPR activation. For example usage of 16F16 (PDI inhibitor) resensitizes tumor cells to imatinib in leukemia cells [132]. Likewise, Doxorubicin or Salubrinal along with bortezomib are more effective against diffuse large B cell lymphoma and hepatoma, respectively. Moreover, in cases of multiple myeloma, toyocamycin has been effective against bortezomib resistance [125, 133].

## CONCLUSIONS AND PERSPECTIVES

The three branches of UPR help tumor cells survive harsh tumor microenvironments and also signal surrounding non-tumor cells to facilitate cancer progression. The contradictory and complex role of each of these transducers in regulating antitumor host responses makes it difficult to put them to practical usage in targeted therapies against cancer. These UPR transducers are regulated by intrinsic timers e.g. prolonged ER stress guides IRE1-dependent decay (RIDD) leading to apoptosis. PERK activation and deactivation are regulated in similar manner. All three branches of UPR may have their own transcriptional factor activity for a single target CHOP, which plays a vital role in deciding the fate of the cell. Silencing a single component of UPR at a time is not a rational therapeutic approach. These targets may require coordination between two branches of the UPR [134, 135]. Currently, experimental models are being used to monitor changes in tumor cells under stress. For example, it has been seen that under hypoxia, the initial phase of eIF2α phosphorylation leads to protein attenuation followed by a transient period of protein synthesis before a permanent reduction in the process of translation [135]. These findings suggest that the effects of future drugs must be time-dependent.

GRP78, which is said to regulate the UPR system, can affect cell apoptosis in different ways by either exhibiting its chaperone activity or by preventing UPR sensor activation and preserving ER calcium homeostasis [136]. Elevated GRP78 levels are usually associated with higher pathologic grade, recurrence, and poor patient survival in cancers of the breast, liver, prostate, and colon [110]. GRP78 assists in tumor progression, spread and drug resistance [110]. Moreover, dormant tumor cells and quiescent tumor cells rely on GRP78 to escape chemotherapy [110]. Several naturally occurring compounds with anticancer activity such as genistein, an active component of soy; epigallocatechin gallate, a component of green tea, and salicyclic acid from plants inhibit either GRP78 expression or its activity [110]. In cancer, versipelostatin, a repressor of GRP78 expression is being used to target the UPR to promote apoptosis or else the usage of proteasomal inhibitors may overload the UPR. Overall, the ideal approach would be to compose both targets without any toxicity. Ideally UPR inhibitors must specifically target the tumor tissue but there is a need to monitor potential toxicities of these inhibitors against B-cells or pancreatic β-cells during future drug discovery efforts [137]. Tissue specific UPR patterns might help to differentiate target tissue and prevent inevitable tumor progression. During chemotherapy, activation of UPR components, particularly PERK, can arrest the cell cycle temporarily but the tumor can regrow once the effect of therapy subsides or cells adapt themselves to stress. Additionally, the inhibition of one branch might result in altered signaling through other branches. As discussed previously, the ICD induction by UPR plays a significant role in the development of novel anticancer strategies. Studies have shown that checkpoint blockade immunotherapy can only be considered for patients when tumors are infiltrated by tumor-infiltrating lymphocytes (TILs) prior to the treatment [138]. In these cases, chemotherapies that do not induce ICD are considered along with immunogenic chemotherapies that may induce UPR response. In conclusion, the capability of the UPR to manage cell fate seems to be a potential cancer therapeutic target. However, contradictory effects of each UPR signaling pathway as well as other confounding factors must be addressed before coming up with a treatment plan involving ER stress regulators.

## Abbreviations

ER: endoplasmic reticulum
UPR: unfolded protein response
PERK: protein kinase (PKR) like ER kinase
IRE1α: inositol-requiring enzyme 1α
ATF6: activating transcription factor 6
GRP78: glucose-regulated protein 78
BiP/GRP78: binding immunoglobulin protein
eIF2: eukaryotic initiation factor 2
ATF4: activating transcription factor 4
CHOP: C/EBP homologous protein
GADD34: growth arrest and DNA damage-inducible
XBP1: X-box binding protein 1
ERAD: ER-associated degradation
JNK: c-Jun N-terminal kinase
ERSE: ER stress response elements
ECM: extracellular matrix
RIDD: IRE1-dependent decay of mRNA
HIF1-α: hypoxia-inducible factor 1 alpha
Ero1: ER oxidoreductin 1
ROS: reactive oxygen species
Nrf2: nuclear factor erythroid 2 [NF-E2]-related factor 2
NF-κB: nuclear factor kappa-light-chain-enhancer of activated B cells
TRAF2: IRE1α–TNF receptor-associated factor 2
ASK1: apoptosis signal regulated kinase
COX-2: cyclooxygenase-2
LTC4: leukotriene C4
EGF: epidermal growth factor
TGF-α: transforming growth factor-α
VEGF: vascular endothelial growth factor
FGF2: fibroblast growth factor 2
IL6: interleukin 6
HCC: hepatocellular carcinoma
EMT: epithelial mesenchymal transition
UPR^mt^: mitochondrial UPR
ERLIN2: endoplasmic reticulum lipid raft associated 2
PDACs: Pancreatic ductal adenocarcinomas
HHV8: Human herpes virus 8
PEL: primary effusion lymphoma
KSHV: Kaposi’s sarcoma herpesvirus
HCV: hepatitis C virus
HSV-1: Human simplex virus 1
SARS CoV: severe acute respiratory syndrome coronavirus
PRRs: pattern recognition receptors
VV: vaccinia viral vector
EBV: Epstein-Barr virus
GM-CSF: granulocyte-macrophage colony-stimulating factor
B-CLL: B-cell chronic lymphocytic leukemia
MAPK: mitogen activated protein kinase
AR: androgen receptor
IL-8: Interleukin-8
M-CSF: macrophage colony-stimulating factor
RANKL: receptor activator for nuclear factor κB ligand
ISRIB: integrated stress response inhibitor
HBV: hepatitis B virus
